# Detection of myocardial edema with diffusion weighted imaging in patients with acute myocarditis

**DOI:** 10.1186/1532-429X-17-S1-P331

**Published:** 2015-02-03

**Authors:** Anna Kociemba, Justyna Rajewska-Tabor, Magdalena Lanocha, Magdalena Janus, Andrzej Siniawski, Katarzyna Karmelita-Katulska, Magorzata Pyda

**Affiliations:** 1 I'st Department of Cardiology, Magnetic Resonance Unit, University of Medical Sciences in Poznan, Poznań, Poland; 2 Department of General Radiology, University of Medical Sciences in Poznan, Poznan, Poland

## Background

Diffusion weighted imaging has been shown to be promising method for myocardial infarction, myocarditis, and myocardial fibrosis imaging. The aim of this study was to evaluate oedema with low b-value (b = 50 sec/mm^2^) diffusion-weighted imaging (DWI) and compare it with routinely used "Lake Louise Criteria" in patients with acute myocarditis.

## Methods

We have analyzed 26 consecutive patients: 22 male, average age: 27 years (range 13-43) with clinical diagnosis of acute myocarditis. The CMR examinations were performed on a 1,5 T scanner using an eight-channel phased-array coil combined with 4-6 elements of spinal coil. All patients underwent assessment of myocardial oedema: T_2_-weighted triple inversion recovery (STIR), T_1_-weighted turbo spin echo pre and post contrast, function (cine Steady State Free Precession) and scar (Late Gadolinium Enhancement). Additionally DWI EPI sequence with b = 50 sec/mm^2^ was acquired before contrast administration. The sequence parameters were as follows: slice thickness 10 mm, repetition time (depending on patient breath cycle) 3-4 s, echo time 78 ms, bandwidth 1,736 Hz/Px. The DWI sequence was ECG-gated and synchronized to the respiratory cycle using PACE technique. For all patients T1 and T2 ratio were calculated and presence of LGE areas were reported. For STIR and DWI contrast between healthy myocardium and edema was calculated as a difference between edematous and normal myocardial muscle divided by standard deviation of image noise.

## Results

We managed to acquired good quality DWI images in all 26 patients, average acquisition time was 120s per slice, distortion artifacts occurred in 5 patients but did not impaired diagnostic value of analyzed images. Increased signal intensity in DWI images occurred in all patients in the area of LGE enhancement and were consistent with areas of increased signal in STIR. All patients met at least two out of three criteria for inflammatory activity and injury. T_2_ ratio was increased (≥ 2) in 24 patients, T_1_ ratio (≥ 4) in 23 cases, all patients had focal non ischemic enhancement in LGE. CNR was higher in DWI than in STIR: 23,8 vs. 17,6 respectively.

## Conclusions

DW EPI is a promising sequence for myocardial oedema detection in patients with acute myocarditis and may be useful in everyday clinical practice.

## Funding

Not applicable.

